# Histiocytosis development and clinical variation through the lens of genomics

**DOI:** 10.1002/path.70081

**Published:** 2026-05-27

**Authors:** Paul G Kemps, Astrid GS van Halteren, Tom van Wezel, Pancras CW Hogendoorn

**Affiliations:** ^1^ Department of Pathology Leiden University Medical Center Leiden The Netherlands; ^2^ Princess Máxima Center for Pediatric Oncology Utrecht The Netherlands; ^3^ Wilhelmina Children's Hospital, University Medical Center Utrecht Utrecht The Netherlands; ^4^ Department of Internal Medicine, Section Allergology & Immunology Erasmus MC University Medical Center Rotterdam Rotterdam The Netherlands; ^5^ Department of Pathology Vrije Universiteit Brussel, Universitair Ziekenhuis Brussel Brussels Belgium

**Keywords:** myeloid cells, mononuclear phagocyte system, hematologic diseases, histiocytosis, leukemia, lymphoma, bone neoplasms, soft tissue neoplasms, protein kinases, sequence analysis

## Abstract

Histiocytic neoplasms are rare haematologic diseases characterised by clonal expansions of cells with a monocyte, macrophage or dendritic cell phenotype. Their clinical manifestations are diverse, ranging from indolent lesions to aggressive systemic disease. Over recent decades, advances in genomic profiling have transformed the biological understanding of these conditions. The discovery of recurrent oncogenic mutations has reframed histiocytoses from primary inflammatory disorders to myeloid neoplasms, with a notable dependence on aberrant mitogen‐activated protein kinase (MAPK) signalling. Novel genetic drivers continue to be uncovered, with many alterations correlating with distinct clinical and pathological phenotypes. Parallel studies have refined the understanding of disease ontogeny, demonstrating that diverse histiocytoses originate from haematopoietic stem/progenitor cells. In Langerhans cell histiocytosis, the differentiation stage of the mutated precursor cell is considered an important — but not the sole — determinant of disease extent and severity. Additional evidence suggests that specific clinical manifestations, such as neurodegenerative disease, may result from somatic mosaicism affecting tissue‐resident macrophages derived from yolk sac progenitors. Collectively, these findings refine histiocytosis diagnosis, risk stratification, disease monitoring and treatment, with robust activity of kinase inhibitors in patients with severe or refractory disease. In this review, we synthesise recent genomic insights into histiocytosis development and variation, while addressing remaining questions and future directions. © 2026 The Author(s). *The Journal of Pathology* published by John Wiley & Sons Ltd on behalf of The Pathological Society of Great Britain and Ireland.

## Introduction

Histiocytic neoplasms are a group of rare diseases characterised by tissue infiltrates of cells with a monocyte, macrophage or dendritic cell phenotype [1, 2, 3]. Historically, these conditions were considered primary inflammatory disorders, with their classification based on distinctive clinical, radiological and pathological features. However, advances in genomic sequencing have revealed that they are driven by a diverse array of somatic mutations, many of which converge on activation of the mitogen‐activated protein kinase (MAPK) signalling pathway [4, 5]. This molecular insight reframed histiocytoses as haematologic neoplasms and sparked a new generation of studies on their developmental origin and phenotypic diversity. In addition, the discovery of molecular disease drivers enabled targeted therapeutic approaches, marking an important transition from empirical to rational treatment [6]. This review synthesises recent insights into histiocytosis development and variation, highlighting how these discoveries have reshaped the understanding, classification and clinical management of these diseases.

### A wide spectrum of diseases

**Figure 1:**
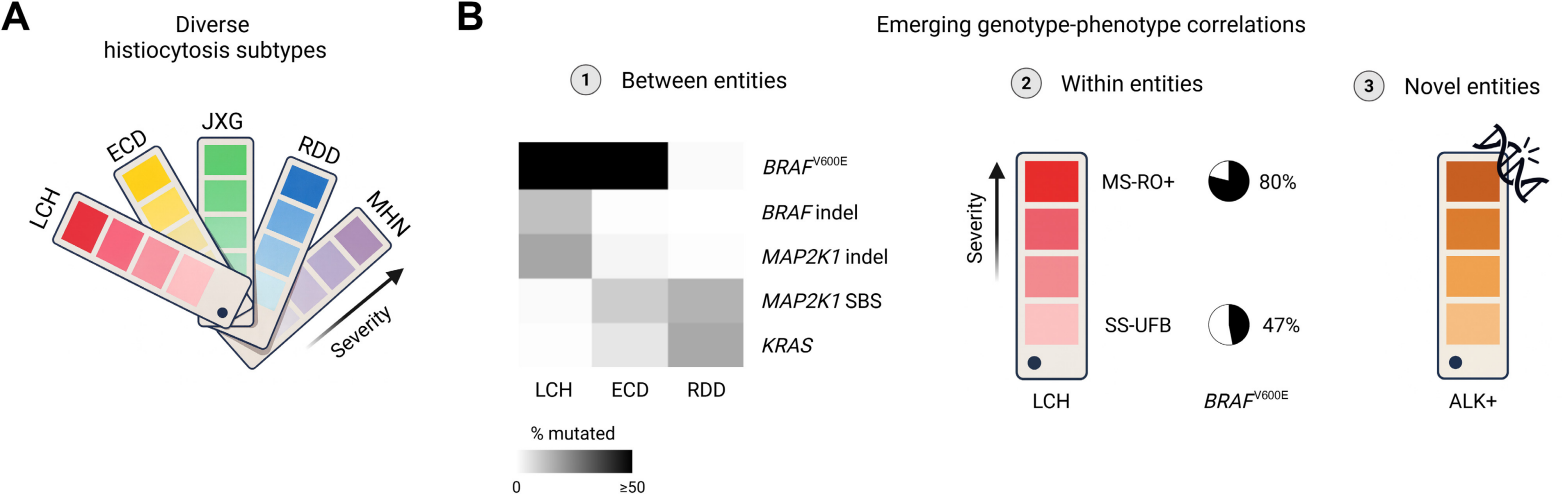
Conceptual framework of histiocytic diseases and emerging clinicogenomic correlations. (A) Histiocytic neoplasms are categorised into defined subgroups, facilitating research and the development of effective treatment approaches. These subgroups can be viewed as distinct colours on a colour swatch fan, with variations in shade reflecting differences in disease severity (darker tone—severe disease; lighter tone—mild disease). (B) Since the discovery of recurrent somatic mutations in histiocytic neoplasms, various clinicogenomic correlations have emerged. First, marked differences in genomic drivers were noted between histiocytic entities. On the left, varying shades of grey depict the differing frequencies of *BRAF*, *MAP2K1* and *KRAS* variants in LCH, ECD and RDD. Second, genotype–phenotype correlations were observed within specific entities. For example, the *BRAF* ^V600E^ mutation is associated with severe disease in children with LCH, occurring in approximately 80% of patients with multisystem disease involving risk organs. Finally, novel entities are beginning to emerge, with ALK‐positive histiocytosis as the first molecularly defined histiocytic entity acknowledged in the WHO classification of haematologic neoplasms. ECD, Erdheim–Chester disease; indel, insertion and/or deletion; JXG, juvenile xanthogranuloma; LCH, Langerhans cell histiocytosis; MHN, malignant histiocytic neoplasm; RDD, Rosai–Dorfman disease; MS‐RO+, multisystem disease with risk organ involvement; SBS, single base substitution; SS‐UFB, single‐system disease with unifocal bone involvement.

Histiocytic neoplasms are remarkably diverse, ranging from indolent neoplasms to highly aggressive cancers (Figure 1A; Table 1) [8, 9, 10, 11]. In the first classification, published in 1987, the diseases were divided into Langerhans cell histiocytosis (LCH), non‐LCH and malignant histiocytic disorders [12]. Although several refinements have been made over the past decades [1, 13], this broad categorisation remains relevant today [14, 15, 16]. LCH is the most studied histiocytosis, occuring in 4.5 per million children younger than 15 years of age and 1.1 per million people aged 15 years or older [17, 18]. LCH is a heterogeneous disease that can affect any organ of the body and ranges from solitary, self‐limiting lesions to life‐threatening disease affecting multiple organ systems. Commonly involved organs are the bone, skin and, in adults, the lungs [17, 19]. A severe form of multisystem LCH tends to affect young children and involves risk organs, including the haematopoietic system, liver and spleen [20, 21]. This subtype is termed ‘high‐risk’ LCH, as it is often refractory to chemotherapy and was historically associated with an increased risk of death [22, 23, 24]. Examples of non‐LCH include Erdheim–Chester disease (ECD) [25], juvenile xanthogranuloma (JXG) [26, 27], Rosai–Dorfman disease (RDD) [28] and indeterminate dendritic cell histiocytosis (IDCH) [29, 30, 31]. ECD and JXG are distinguished based on clinical and radiographic findings, as they have a similar histomorphology and shared immunophenotype [32, 33]. Yet, the histiocytes in ECD can be scarce and obscured by fibrosis, particularly in bone biopsies [34]. Moreover, foamy histiocytes and Touton giant cells may be rare or absent. The malignant histiocytic neoplasms (MHNs) include histiocytic sarcoma (HS), interdigitating dendritic cell sarcoma (IDCS) and Langerhans cell sarcoma (LCS) [1, 2, 3]. These are immunophenotypic subtypes of histiocytic neoplasms displaying anaplastic histology [1]. A recent study, however, showed that MHNs comprise four major subgroups [35]. These subgroups parallel the lineage differentiation of monocytes, macrophages, dendritic cells and Langerhans cells; accordingly, they parallel the lineage differentiation of histiocytic neoplasms with low‐grade histology [35]. The disease can present in isolation, termed ‘primary MHN’, or may follow another haematologic neoplasm, termed ‘secondary MHN’ or ‘MHN with an associated haematologic neoplasm’ [1]. Interestingly, a substantial proportion of patients exhibit clinical and/or pathological features of multiple histiocytoses. For example, 10–15% of patients with ECD also have LCH lesions, and both diseases are associated with the same clinical complications (i.e., arginine vasopressin deficiency and neurodegenerative disease) [1, 36, 37]. Other histiocytosis combinations are also encountered [38, 39]. Therefore, histiocytic neoplasms are best considered a spectrum of diseases, crossing the boundary between benign and malignant neoplasms.

**Table 1:** Pathologic features of histiocytic neoplasms.

							MHN*		
	LCH	IDCH	ECD	JXG	RDD	ALK+	LCS	HS	IDCS
Immunophenotypic features									
PU.1	+	+	+	+	+	+	+	+	+
CD1a	+	+	−	−	−	−	+	−	−
CD207 (Langerin)	+	−	−	−	−	−	+	−	−
S100	+	+/−	−/+	−/+	+	−/+	+	−/+	+
CD163	−	−/+	+	+	+/−	+	+/−	+	+/−
ALK^†^	−	−	−	−	−	+	−	−	−
Histological features									
Xanthomatous histiocytes	−	−	+/−	+	−	−/+	−	−	−
Touton giant cells	−	−	−/+	+	−	−/+	−	−	−
Emperipolesis	−	−	−/+	−/+	+	−/+	−	−/+	+/−
Anaplasia	−	−	−	−	−	−	+	+	+

ALK+, ALK‐positive histiocytosis; ECD, Erdheim–Chester disease; HS, histiocytic sarcoma; IDCH, indeterminate dendritic cell histiocytosis; IDCS, interdigitating dendritic cell sarcoma; JXG, juvenile xanthogranuloma; LCH, Langerhans cell histiocytosis; LCS, Langerhans cell sarcoma; MHN, malignant histiocytic neoplasm; RDD, Rosai−Dorfman disease.

* In contrast to low‐grade histiocytic neoplasms, the immunophenotype of MHNs is often heterogeneous with disorderly lineage differentiation. This is particularly true for MHNs with a Langerhans cell phenotype, which often demonstrate co‐expression of macrophage or monocyte markers.

^†^ ALK expression is occasionally observed in histiocytic neoplasms without *ALK* fusions. These can be distinguished from *ALK*‐rearranged cases by the presence of nuclear ALK expression on immunohistochemistry [7].

### Recognition as haematologic neoplasms

Based on their benign histologic appearance, dominant inflammatory infiltrate and occasional spontaneous regression, LCH and non‐LCH were long considered inflammatory disorders. In 1994, strong support for a neoplastic origin of LCH was provided by two independent research groups, revealing clonality of LCH‐associated histiocytes by demonstrating non‐random X chromosome inactivation [40, 41]. However, clonality alone is insufficient for the designation of a disease as a neoplasm. In 2008, a *TPM3*::*ALK* fusion and *NOTCH1* mutations were reported in two cases of histiocytosis, but these alterations were not demonstrated to be recurrent [42, 43]. Moreover, no gross chromosomal abnormalities were identified in LCH using diverse molecular technologies [44]. In 2010, a breakthrough came with the discovery of somatic *BRAF* ^V600E^ mutations in about half of LCH cases [45]. This discovery was made possible by advances in genomic technology, which enabled the simultaneous assessment of mutations in hundreds of genes in fragmented DNA isolated from formalin‐fixed paraffin‐embedded tissues. This technology unlocked pathology archives around the world to genomic investigation, which quickly led to the recognition of diverse histiocytoses as haematologic neoplasms [4].

## Genomic variation linked to histiocytosis diversity

### Genomic variation between histiocytic entities

Soon after the seminal discovery of *BRAF* ^V600E^ in LCH [45], various research groups revealed that this mutation is also highly prevalent in ECD [46] and JXG of the central nervous system (CNS) [33, 47]. However, the mutation was only rarely detected in other histiocytoses [46, 48, 49, 50, 51]. Similarly, other kinase alterations were found to be enriched in specific histiocytosis subgroups, such as *BRAF* exon 12 deletions in LCH [49, 51, 52], *ETV3*::*NCOA2* fusions in IDCH [53] and *NTRK1* fusions in cutaneous JXG [51, 54, 55, 56]. Moreover, *MAP2K1* mutations differed by histiocytosis subtype, with deletions being common in LCH and single base substitutions prevailing in ECD and RDD (Figure 1B) [57]. These findings demonstrate that histiocytic neoplasms exhibit distinct genomic landscapes, including JXG and ECD despite their pathological similarity.

### ALK‐positive histiocytosis: the first molecularly defined histiocytic neoplasm

Perhaps the clearest example of the intimate link between genetic driver alterations and histiocytosis phenotypes is ALK‐positive histiocytosis. This disease was first described in 2008 and recently established as a distinct histiocytic entity often characterised by *KIF5B*::*ALK* fusions and neurologic involvement [42, 58, 59]. Previously, the disease had sometimes been reported as ECD or JXG [48, 49, 51, 60, 61, 62, 63], yet many clinical and radiological characteristics of ECD were not observed, and the clinical phenotype was also markedly different from (systemic) JXG without *ALK* rearrangements [59, 64]. Moreover, ALK‐positive histiocytosis exhibited a female predilection, whereas ECD and JXG are more common in males [26, 27, 33, 65, 66, 67, 68]. Based on these unique features, ALK‐positive histiocytosis was recognized as a distinct histiocytic entity in the 5th World Health Organization Classification of Haematolymphoid Tumours and the International Consensus Classification of Mature Lymphoid, Histiocytic, and Dendritic Cell Tumours (Figure 1B) [2, 3]. Thereby, ALK‐positive histiocytosis set the precedent for molecular (sub)classification of histiocytic neoplasms, guiding optimal diagnosis, staging and personalised treatment of patients.

### Genotype–phenotype correlations within histiocytic entities

**Figure 2:**
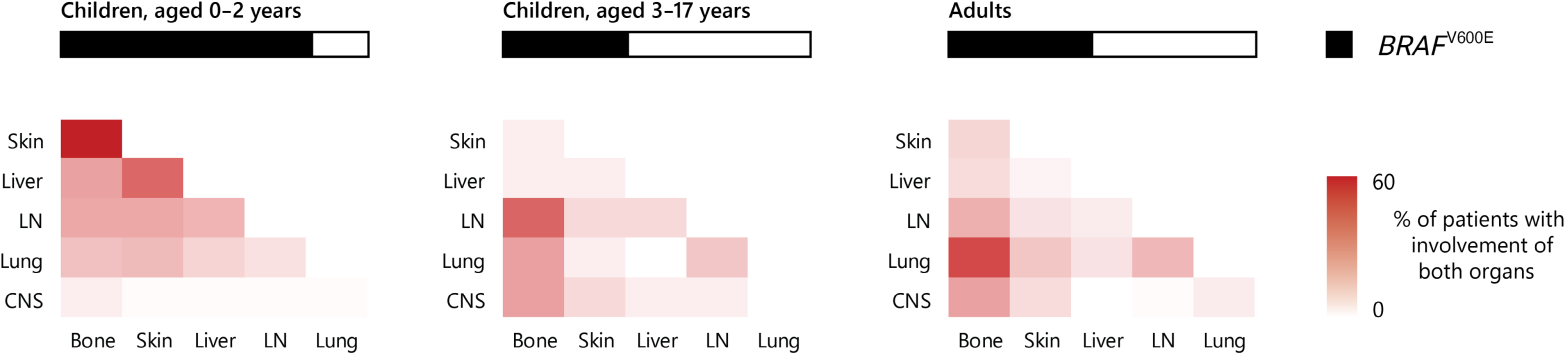
Distinct patterns of organ involvement in children and adults with multisystem LCH. Within each group of patients with multisystem LCH, the percentage of patients with *BRAF* ^V600E^ is indicated by a black bar. Below this bar, the percentage of patients with disease involvement of two organs is indicated by shades of red, with 60% in dark red and 0% in white. Based on data of two international cohort studies of children and adults with LCH [69, 73], including 67 children aged 0–2 years at diagnosis of multisystem LCH, 22 children aged 3–17 years at diagnosis of multisystem LCH, and 58 adults with multisystem LCH. LCH, Langerhans cell histiocytosis; LN, lymph node; CNS, central nervous system.

Within histiocytosis subgroups, different driver mutations were also found to correlate with distinct clinical phenotypes. In children with LCH, the *BRAF* ^V600E^ mutation strongly correlated with severe disease and inferior outcomes (Figure 1B) [21, 69, 70]. In contrast, *MAP2K1* mutations were associated with single‐system bone LCH, whereas *BRAF* exon 12 deletions appeared to correlate with lung involvement [69, 71, 72]. Interestingly, *BRAF* ^V600E^ did not correlate with multisystem disease in adults with LCH [73], showing that insights in children cannot simply be adopted to the adult population. Clinically, multisystem LCH in adults is also substantially different from multisystem LCH in young children, with a distinct pattern of organ involvement (Figure 2). Instead of *BRAF* ^V600E^, *BRAF* exon 12 deletions were recently shown to correlate with multisystem disease in adults with LCH, wherein they specifically associate with hepatic, vulvar and pituitary involvement [52, 74, 75]. In ECD, a multitude of studies have revealed that *BRAF* ^V600E^ correlates with sinus [76], cardiac [66, 77], aortic [78], CNS [66, 79] and perirenal [67] involvement, as well as with mesenteric panniculitis [80] and arginine vasopressin deficiency [66]. Moreover, the mutation was found to be highly prevalent in patients with mixed ECD/LCH [36, 37, 39], whereas *MAP2K1* mutations were common in patients with ECD accompanied by RDD, who often exhibited testicular lesions [38]. These mutation‐associated clinical subtypes of ECD were also identified using unsupervised clustering [81]. In JXG, we and others demonstrated recurrent *CLTC*::*SYK* fusions and *CSF1R* mutations in children younger than 2 years of age who presented with soft tissue tumours [82, 83]. Although *CSF1R* mutations have been occasionally reported in other histiocytic neoplasms [51, 84, 85], the *CLTC*::*SYK* fusion appears specific to this rare manifestation of JXG [82, 83]. Interestingly, the fusion of *SYK* to another gene (*ETV6*) seems tightly linked to a systemic myeloid neoplasm with histiocytic skin lesions among adults [86, 87, 88, 89, 90, 91, 92, 93]. Thus, the fusion partner is also relevant. Accordingly, the *KIF5B*::*ALK* fusion is frequent in ALK‐positive histiocytosis [58, 59], but rarely detected in other *ALK*‐rearranged neoplasms [94, 95]. Similarly, *NCOA2* fusions are oncogenic drivers in various cancers [96, 97, 98, 99], but the *ETV3*::*NCOA2* fusion seems histiocytosis specific. The selection of fusion partners is primarily determined by fusion protein stability and active transcription of fusion partner genes [100]. As ETV3 and ETV6 are transcriptional repressors that enable monocyte differentiation into dendritic cells [101], their expression in myeloid precursors may explain their involvement in histiocytosis‐associated gene fusions. Finally, Egan *et al* revealed a distinct molecular subgroup of primary histiocytic sarcoma characterised by *NF1*/*PTPN11* alterations and a predilection for the gastrointestinal tract [102], which was confirmed by another study [103]. Taken together, these studies have convincingly demonstrated that genetic drivers are intimately linked to phenotypic diversity across histiocytosis subgroups.

## Revised ontogeny of histiocytic neoplasms

### Haematopoietic progenitors as the cell of origin

Prior to the discovery of oncogenic mutations, the assumption that histiocytic diseases originate from mature macrophages or dendritic cells had already been questioned. Gene expression profiling of CD207^+^ histiocytes from LCH lesions had revealed a transcriptional profile substantially different from epidermal Langerhans cells and more closely aligned with that of myeloid precursor cells [5, 104, 105, 106]. The subsequent identification of somatic mutations allowed researchers to track the neoplastic clone in the haematopoietic system and define the cellular origins of histiocytic neoplasms in more detail [106, 107, 108, 109, 110, 111, 112]. In bone marrow of patients with systemic LCH and/or ECD, the *BRAF* ^V600E^ mutation could be identified in CD34^+^ haematopoietic stem/progenitor cells (HSPCs) [70, 107, 108, 109, 113]. The percentage of HSPCs harbouring the mutation was typically very low (<1%), indicating that *BRAF* ^V600E^ does not confer a powerful selective advantage to bone marrow progenitors [70, 107, 108, 109, 113]. This notion is supported by *in vitro* and *in vivo* studies [114, 115, 116, 117]. Mutated CD34^+^ cells of patients with ECD demonstrated clonal potential in colony‐forming assays and formed histiocytic lesions characteristic of ECD in xenograft mouse models [109, 113, 118]. Similarly, murine xenografts of unsorted bone marrow from children with high‐risk LCH exhibited a disease very similar to human LCH [119]. Finally, LCH‐like lesions were observed when *BRAF* ^V600E^ or a *MAP2K1* mutation were artificially introduced in human CD34^+^ cells that were transplanted into immunocompromised mice [120]. Together, these findings provided functional evidence that mutated HSPCs from patients with histiocytosis can drive their disease.

**Figure 3:**
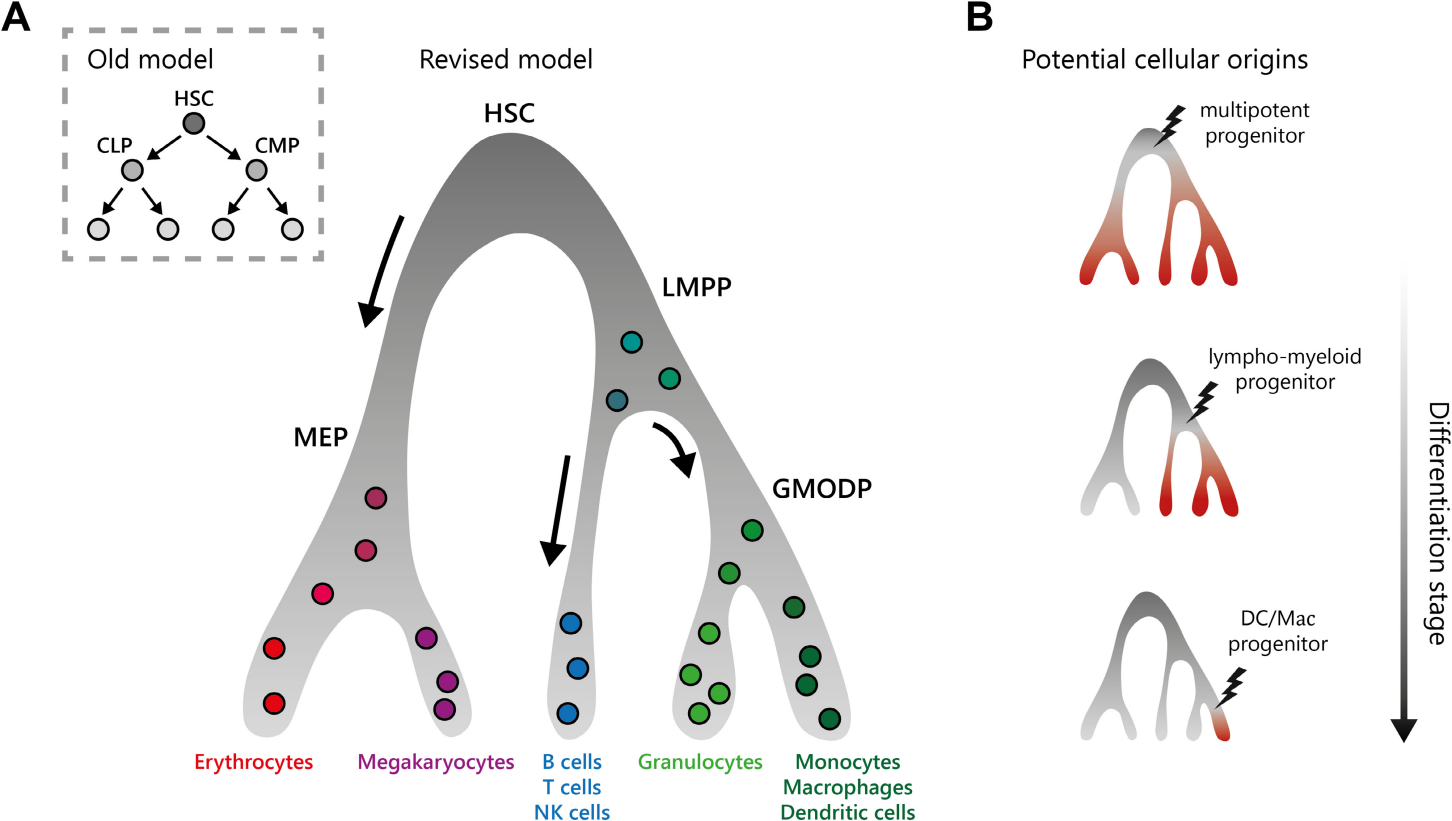
Contemporary model of human haematopoiesis and potential cellular origins of histiocytic neoplasms. (A) Schematic representations of the old and revised models of human haematopoiesis. The revised model is based on single‐cell studies and indicates a continuum of differentiation, rather than binary fate decisions at discrete stages of haematopoietic differentiation. (B) The cellular origin of histiocytic neoplasms may lie at any point across the continuum of progenitor states, ranging from multipotent progenitors at the apex, to lympho‐myeloid progenitors that lack erythroid differentiation potential, to more committed dendritic cell/macrophage progenitors. CLP, common lymphoid progenitor; CMP, common myeloid progenitor; GMODP, granulocyte/monocyte/osteoclast/dendritic cell progenitor; HSC, haematopoietic stem cell; LMPP, lymphoid‐primed multipotent progenitor; MEP, megakaryocyte‐erythroid progenitor; NK, natural killer. Panel A is based on an illustration by Laurenti *et al* [124].

Driver mutations in *BRAF*, *MAP2K1* and *KRAS* were also detected in peripheral blood mononuclear cells (PBMCs) of patients with diverse histiocytic neoplasms, although often at low frequencies [70, 106, 107, 108, 109, 111, 112, 121]. Detailed analysis of the cellular distribution of mutant alleles revealed significant enrichment in monocytes and myeloid dendritic cells, although mutations were also regularly detected in lymphoid subsets [70, 106, 107, 108, 110, 112, 121]. Because circulating mutated cells were consistently identified in children with high‐risk LCH and often not in those with single‐system disease, it was hypothesised that the clinical extent and severity of LCH are defined by the stage of differentiation of the precursor cell in which the driver mutation arises [70, 107, 122, 123]. Together with evolving concepts of human haematopoiesis—in which differentiation is understood as a dynamic continuum of progenitors rather than a rigid hierarchy [124]—this offered a framework for understanding the diverse clinical phenotypes produced by the same driver mutation (Figure 3). In this ‘misguided myeloid differentiation’ model, mutations in bone marrow HSPCs give rise to high‐risk LCH, whereas the same mutations in more committed, circulating or tissue‐restricted, myeloid precursor cells give rise to low‐risk LCH [123]. This model of LCH ontogeny was supported by mouse experiments, which demonstrated that expression of *BRAF* ^V600E^ in bone marrow‐resident dendritic cell progenitors led to an aggressive LCH‐like disease, whereas expression of the same mutation in more mature CD207^+^ dendritic cells led to a less severe LCH‐like phenotype [107]. The model, however, appears somewhat oversimplified, as recent studies have shown that a substantial proportion of patients with single‐system LCH carry mutated cells in the blood [70, 108, 110, 125, 126, 127, 128]. Similarly, mutations driving other histiocytoses confined to a single organ—such as skin‐limited xanthogranulomas—could be traced to circulating hematopoietic cells, including CD34^+^ progenitors [121]. Using single‐cell multi‐omics, the presence and effect of genetic variants in different haematopoietic populations could be investigated further [129]. These techniques have been pioneered in studies of myeloproliferative neoplasms, in which a handful of driver mutations also lead to diverse clinical phenotypes [130]. As the proportion of mutant cells in the blood and bone marrow is generally low in histiocytosis, we hypothesize that the tissue provides the necessary environment for the clonal outgrowth of these cells.

### Clonal haematopoiesis and myeloid cancers among adult patients

A growing body of evidence suggests a role for clonal haematopoiesis (CH) in the development of histiocytic neoplasms among adults [113, 131]. CH refers to the clonal outgrowth of haematopoietic cells, which is characterised by the overrepresentation of blood cells derived from a specific haematopoietic stem cell (HSC) [132]. This is often caused by genetic alterations that confer a fitness advantage, allowing the mutated stem cell and its progeny to expand [132]. Thus, leukaemia can be considered an extreme example of CH [132]. Yet, the term is generally used to refer to the premalignant state [2, 133], which is formally termed clonal haematopoiesis of indeterminate potential (CHIP). Commonly associated genetic alterations are loss‐of‐function or truncating mutations in epigenetic regulator genes *ASXL1*, *DNMT3A* and *TET2* [134]. The condition is considered a phenomenon of aging because its prevalence increases dramatically with age [134]. It is associated with an increased risk of haematologic malignancies and cardiovascular disease [135, 136, 137], while it may reduce the risk of Alzheimer's disease [138]. Through next‐generation sequencing of bone marrow samples, Cohen‐Aubart *et al* demonstrated a high frequency (43%) of CH in 120 patients with ECD [113]. CH was significantly enriched in patients with ECD and an additional haematologic malignancy; one‐third of patients with CH had such a malignancy (16 myeloid; 1 lymphoid) compared with only 3% of patients without CH. By analysing colonies derived from single CD34^+^ cells, the authors showed that ECD driver mutations can be acquired by pre‐existent, *TET2*‐mutated progenitors [113]. In addition, an increasing number of studies have revealed identical somatic mutations in histiocytic neoplasms and associated myeloid malignancies [14, 68, 109, 139, 140], supporting a common clonal origin. These findings shed new light on the molecular underpinnings of histiocytic neoplasms among adults and suggest that CH may contribute to the development of additional, clonally related myeloid neoplasms in this population [141].

### Histiocytic neoplasms secondary to lymphoid malignancies

Clonal relationships between lymphoid cancers and subsequent histiocytic neoplasms were first demonstrated in the early 2000s through the detection of identical immunoglobulin or T‐cell receptor gene rearrangements [142, 143, 144, 145]. As these rearrangements represent molecular hallmarks of B‐ or T‐lineage commitment [146, 147], these findings suggested some form of transdifferentiation of lymphoid‐committed precursor cells to the myeloid lineage, potentially requiring the dedifferentiation of these cells as an intermediate step [145, 148]. *In vitro* studies have revealed that such conversion to the myeloid lineage can be driven by altered expression of lineage‐specific transcription factors, such as overexpression of the myeloid transcription factor C/EBPα and loss of the master regulator of B‐cell commitment Pax5 [149, 150, 151, 152, 153, 154, 155]. Consistent with these findings, several patients with *PAX5*‐mutated B‐cell acute lymphoblastic leukaemia (B‐ALL) and secondary histiocytic sarcoma have been reported [68, 156, 157], supporting that loss of Pax5 predisposes precursor B‐cells to a myeloid lineage switch [158, 159, 160, 161, 162]. In T‐cell acute lymphoblastic leukaemia (T‐ALL), secondary histiocytic neoplasms are overrepresented in the molecular subgroup defined by *SPI1* (PU.1) fusions [163]. The leukemic blasts of these patients exhibit enriched expression of dendritic cell‐associated genes and may be more amenable to myeloid conversion [163]. The ultimate drivers of the lineage switch remain to be elucidated but may involve secondary hits in genes of the MAPK signalling pathway, as these have been frequently detected in secondary histiocytic neoplasms [68, 140]. In addition, it may be triggered by selective pressure induced by lymphoid lineage‐directed therapies [164, 165, 166, 167, 168, 169, 170]. Supporting this notion, an increasing number of patients have been described with histiocytic sarcoma secondary to B‐ALL or B‐cell lymphoma after treatment with blinatumomab and/or CD19‐directed chimeric antigen receptor (CAR) T‐cells [171, 172, 173, 174]. With the expanding use of these novel agents, it will be essential to further elucidate this phenomenon and develop effective strategies to prevent or counteract it.

### Haematopoietic origin of histiocytosis‐associated neurodegeneration

The discovery of *BRAF* ^V600E^ also reshaped the understanding of histiocytosis‐associated neurodegeneration [175, 176], which is strongly associated with this mutation [66, 177, 178]. Neurodegenerative (ND)‐LCH often presents as a late complication, arising years after a patient is presumed to be cured from systemic LCH [177, 179, 180]. Clinically, it generally manifests as a progressive cerebellar syndrome [181, 182, 183]; radiologically, it is characterised by non‐tumorous lesions in the cerebellum, basal ganglia and/or brainstem [181, 182, 183]. Initially, ND‐LCH was thought to represent a paraneoplastic or autoimmune phenomenon because brain lesions typically lacked CD1a^+^ CD207^+^ cells and were characterised by a dominant T‐cell infiltrate [182, 184, 185]. However, McClain *et al* revealed circulating *BRAF* ^V600E^‐mutated cells in patients with ND‐LCH, in the absence of systemic lesions. In addition, they demonstrated *BRAF* ^V600E^ expression in perivascular monocytes/macrophages enriched at sites of active demyelination [6, 175]. In a follow‐up study, *BRAF* ^V600E^‐mutated myeloid cells were shown to break through the blood–brain barrier and infiltrate the brain in a mouse model of ND‐LCH, driving a neurodegenerative disease similar to that observed in humans [176]. Together, these data support that histiocytosis‐associated neurodegeneration can arise from mutated haematopoietic cells, clonal to (prior) systemic lesions [186].

### Prenatal origin from yolk sac progenitors

**Figure 4:**
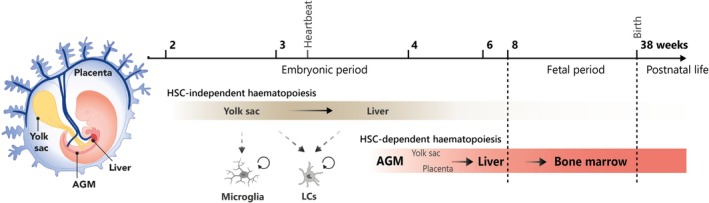
Prenatal origins of tissue‐resident macrophages, including microglia and Langerhans cells. Tissue‐resident macrophages like microglia and Langerhans cells are derived from HSC‐independent progenitors that originate from the yolk sac and eventually seed the liver [187]. HSCs emerge in the AGM region and then migrate through the yolk sac and placenta before they colonise the liver. During the second trimester, HSCs transition to the bone marrow [188]. Shown on the left is a human embryo at Cargenie stage 15 (5 weeks). AGM, aorta‐gonad‐mesonephros; HSC, haematopoietic stem cell; LC, Langerhans cell. Adapted from Calvanese *et al* with permission from the publisher (Elsevier) [188].

Introducing a final layer of complexity to the ontogeny of histiocytic neoplasms, the origins of these diseases may also lie in embryonic or fetal haematopoiesis. Prenatal haematopoiesis is a complex process, characterised by temporal waves of blood formation at different anatomical sites (Figure 4) [188]. The first blood cells do not arise from HSCs but come from primitive haematopoietic progenitors [188]. These progenitors originate outside the embryo from the yolk sac and subsequently seed the liver [188]. It is these early progenitors that give rise to tissue‐resident macrophages, including microglia in the brain and Langerhans cells in the epidermis [187, 189, 190, 191, 192]. These two cell types populate the respective tissues before birth and subsequently self‐maintain throughout life under steady state conditions [193, 194, 195]. At 4–5 weeks after conception, HSCs arise in the embryo and gradually take over haematopoiesis [188]. In theory, a driver mutation could arise at any point during prenatal haematopoiesis and cause histiocytic disease. This includes a mutation in a bone marrow HSC, but also a mutation in an HSC‐independent primitive haematopoietic progenitor. In the context of LCH, it has been hypothesised that congenital, self‐resolving, skin‐limited LCH may arise from a mutation in such a primitive Langerhans cell precursor from the yolk sac or liver [196]. Similarly, it has been proposed that histiocytosis‐associated neurodegeneration may be caused by a mutation in a yolk sac‐derived microglia precursor [197]. This hypothesis is substantiated by a mouse model, in which enforced mosaic expression of *BRAF* ^V600E^ in yolk sac‐derived erythromyeloid progenitors led to a clonal expansion of tissue‐resident macrophages, including microglia in the brain. The mice subsequently developed a late‐onset neurodegenerative disorder—in the absence of systemic disease, which relied on ERK‐activated microglia.

In a recent follow‐up study, the authors undertook a comprehensive study of brain samples of human histiocytosis patients with (*n* = 4) and without (*n* = 4) clinical neurodegeneration. These patients had been diagnosed with systemic LCH and/or ECD between two and 26 years earlier [198]. Surprisingly, they found *BRAF*‐mutated cells with characteristics of microglia in the brains of patients with and without neurological symptoms. The presence of neurological symptoms was associated with a longer evolution of the histiocytic disease and a larger size of *BRAF*‐mutated clones in the brain. To investigate the origin of these mutated cells, Vicario *et al* assessed whether *BRAF* ^V600E^ and other single nucleotide variants identified in these cells were also present in matched blood or bone marrow cells. In multiple patients, the authors did not detect these mutations in the blood or bone marrow; therefore, they hypothesised that the *BRAF*‐mutated cells in the brains of these patients may have originated from yolk sac‐derived microglia precursors. However, it is also possible that the *BRAF*‐mutated clones had already disappeared from the circulation at the time of blood or bone marrow collection. Accordingly, one of these patients was analysed by single nuclei genotyping, which revealed that the *BRAF*‐mutated cells in the brain harboured the same *TET2* and *DNMT3A* mutations as were detected in the bone marrow. This indicates that the *BRAF*‐mutated clone derived from CH in this patient. Thus, the mutated cells have likely infiltrated the brain earlier, where they adopted a microglia‐like phenotype and transcriptional program and exhibited long‐term persistence. Similarly, *BRAF*‐mutated cells appear capable of forming long‐lived macrophage populations in the dermal layer of the skin of patients with histiocytosis [199]. In the brain, the mutated cells appear to slowly disrupt tissue homeostasis. This notion is substantiated by a recent study showing that *BRAF*‐mutated microglia‐like cells generated from induced pluripotent stem cell (iPSC)‐derived CD34^+^ progenitors cause neurodegeneration in cocultures with iPSC‐derived neurons [200]. A haematopoietic origin of histiocytosis‐associated neurodegeneration also fits with the typical pattern of the disease, whereby patients first develop systemic LCH and/or ECD and then go on to develop neurodegeneration [177, 185]. If histiocytosis‐associated neurodegeneration would be driven by mutated microglia, the relationship with prior systemic disease would be difficult to explain [176]. To further investigate this issue, the somatic mutation landscapes of *BRAF*‐mutated cells from the brain could be compared to *BRAF*‐mutated histiocytes from (prior) lesions in other organs. In addition, phylogenetic studies could time driver mutations relative to conception, positioning them within the sequential waves of developmental haematopoiesis [201].

## The path from oncogene activation to histiocytosis development and inflammation

**Figure 5:**
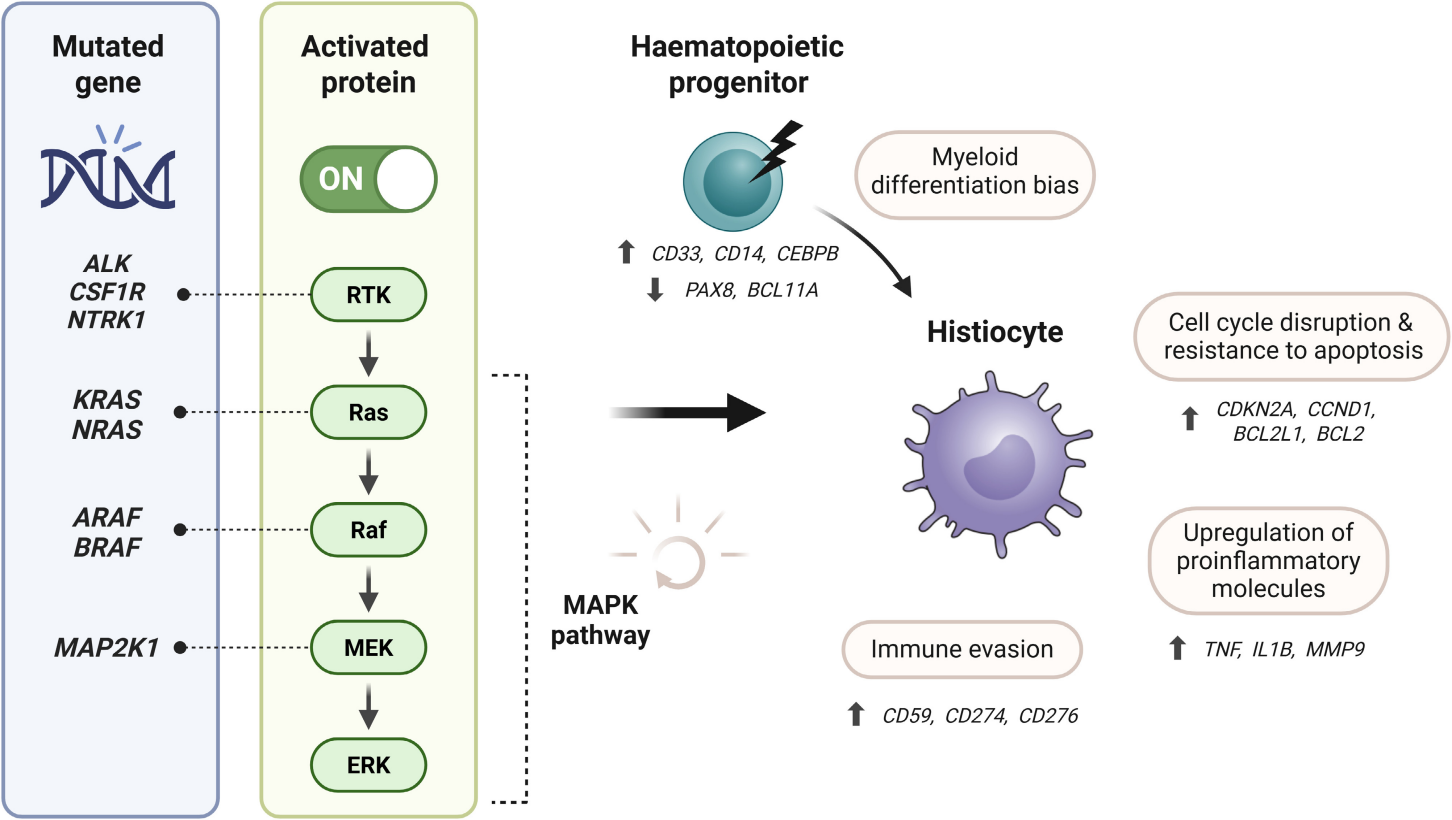
Pathogenesis of histiocytic neoplasms. Somatic mutations in genes encoding for receptor tyrosine kinases or downstream kinases lead to constitutively activated proteins. These proteins signal through the MAPK signalling pathway, which consists of a cascade of kinases, in which Ras activates Raf, Raf activates MEK, and MEK activates ERK. ERK enters the nucleus and affects the transcription of various genes involved in cellular differentiation, survival and immune modulation. For example, MAPK pathway activation driven by *BRAF* ^V600E^ leads to a myeloid differentiation bias of haematopoietic progenitors, with transcriptional upregulation of genes involved in monocyte and dendritic cell differentiation and concurrent downregulation of genes that promote lymphoid fate. *BRAF* ^V600E^‐mutated histiocytes exhibit altered expression of cell cycle regulators and upregulation of genes encoding anti‐apoptotic proteins BCL‐xL and BCL2. Finally, *BRAF* ^V600E^ expression leads to the production of various proinflammatory molecules early during myeloid differentiation and increases the expression of immune checkpoint molecules, such as *CD274* (PD‐L1), which are involved in immune response suppression. For each described effect of *BRAF* ^V600E^, several examples of up‐ or down‐regulated genes are indicated. ERK, extracellular signal‐regulated kinase; MAPK, mitogen‐activated protein kinase; MEK, mitogen‐activated protein kinase; RTK, receptor tyrosine kinase. Created with BioRender.com.

How MAPK pathway activation leads to histiocytosis development and associated inflammation remains a field of active investigation. Much of the current evidence is derived from mechanistic studies of LCH and ECD and may not fully apply to the other histiocytic neoplasms. The available data indicate that histiocytosis‐associated driver mutations induce a myeloid differentiation bias in haematopoietic progenitors (Figure 5). Enforced expression of the *BRAF* ^V600E^ mutation in mouse or human HSPCs enhanced their differentiation towards the mononuclear phagocyte lineage [107, 115, 116, 117, 120, 200]. This process seemed to be mediated by altered expression of key transcription factors [200]. Similarly, myeloid skewing was observed after enforced expression of a *MAP2K1* deletion in mice [202]. In line with these findings, CD34^+^ HSPCs from patients with LCH exhibited increased expression of genes involved in macrophage or dendritic cell commitment [115]. The lineage bias of these precursor cells is reflected in the peripheral blood, as patients with histiocytosis regularly have increased frequencies of circulating monocytes compared with healthy controls [108, 203, 204]. Several studies have also linked *BRAF* ^V600E^ expression to cell cycle arrest, with increased expression of cell cycle regulators like *CDKN2A* (p16), *TP53* (p53) and *CCND1* (cyclin D1) [115, 116, 205, 206], although these findings were not reproduced in a recent study [200]. A more consistent observation is that *BRAF* ^V600E^‐mutated macrophages and dendritic cells exhibit resistance to apoptosis via enhanced expression of anti‐apoptotic proteins—including BCL‐xL (encoded by *BCL2L1*) and BCL2 [104, 114, 200, 207]. Coupled with *BRAF* ^V600E^‐induced suppression of CCR7 expression, which impairs dendritic cell migration to draining lymph nodes, this results in the accumulation of neoplastic histiocytes in peripheral tissues [104, 114, 205, 208]. *BRAF* ^V600E^ also directly contributes to the characteristic inflammation in histiocytosis through the upregulation of various pro‐inflammatory molecules early during myeloid differentiation [114, 115, 116, 200, 209, 210]. In LCH, the most differentiated cells express genes linked to destructive inflammatory behaviour, such as those encoding matrix metalloproteinases (*MMP9* and *MMP12*) or genes associated with osteoclast differentiation [211]. This suggests that these cells may be particularly involved in the observed tissue destruction, including osteolysis and fibrosis [211]. Some researchers have proposed that the cellular state and secretory phenotype induced by *BRAF* ^V600E^ is characteristic of oncogene‐induced senescence [115, 116]. Others have attributed the inflammatory phenotype to the intrinsic nature of the disease, given that the upregulated molecules are naturally enriched in cells of the mononuclear phagocyte lineage [200]. Interestingly, the secretome of *BRAF* ^V600E^‐mutated progenitors also affects their non‐mutated counterparts, skewing their differentiation and promoting their release of proinflammatory cytokines [115, 116]. A key determinant of this paracrine transmission is TNF‐α produced by the *BRAF‐*mutated cells [116, 210]. The importance of aberrant signalling in non‐mutated cells should not be overlooked. After all, the proportion of mutated cells in histiocytosis lesions is generally low [212]. Moreover, risk organ involvement in LCH is typically not caused by diffuse infiltration by LCH cells but rather a manifestation of systemic inflammation [213, 214]. Finally, accumulating evidence points to immunosuppressive effects of the *BRAF* ^V600E^ mutation, which is correlated with reduced (CD8^+^) T‐lymphocyte infiltration in both LCH and ECD [215, 216, 217]. Consistent with this concept, blocking the effects of the mutation through MAPK pathway inhibition led to an increase in CD8^+^ T‐cell infiltration in a mouse model of LCH [216]. Neoplastic histiocytes seem to upregulate immune checkpoint molecules, such as PD‐L1 and PD‐L2 [205, 216, 217, 218, 219, 220], whereas lesional T‐cells display increased expression of corresponding inhibitory receptors and exhibit impaired effector functions [216]. Taken together, these findings suggest that MAPK pathway activation not only transforms myeloid progenitors but also establishes a suppressive immune microenvironment that enables histiocytosis persistence. Accordingly, histiocytic neoplasms can be regarded as a pathologic combination of oncogenesis and immune dysregulation [221].

## Clinical implications

The genomic discoveries in histiocytosis have already changed clinical practice. Molecular testing now forms an important component of diagnostic evaluation, providing guidance in cases with ambiguous histopathology. This includes distinguishing neoplastic histiocytoses from reactive proliferations, which can be notoriously difficult. Moreover, genetic analysis of bone marrow, blood, urine or cerebrospinal fluid can assist in histiocytosis diagnosis in patients with poorly accessible lesions [222]. Some genetic drivers correspond to specific histiocytosis phenotypes, enabling a more refined diagnosis and a better understanding of the likely clinical trajectory. But above all, genomic insights have informed novel approaches to risk stratification, disease monitoring and treatment — as detailed below.

### Molecular disease detection in blood or bone marrow

For many decades, first‐line treatment in paediatric LCH was solely based on clinical presentation, with no molecular marker used in risk stratification [211]. In our international retrospective study, lesional *BRAF* ^V600E^ status was not a significant prognostic factor independent from disease extent, limiting its utility in this context [69]. However, molecular analysis of peripheral blood or bone marrow appears promising. In a Chinese study of children with LCH, the detection of *BRAF* ^V600E^ in plasma‐derived cell‐free DNA (cfDNA) at diagnosis correlated with reduced progression‐free survival, including in the subgroup of patients with single‐system LCH [223]. Accordingly, the detection of *BRAF* ^V600E^ in plasma or PBMCs was an independent prognostic factor in a follow‐up study [126]. These findings were confirmed by a recent, much larger study from the United States [70]. In addition, the latter study demonstrated that *BRAF* ^V600E^ detection in PBMCs at diagnosis was associated with a higher risk of developing ND‐LCH [70]. Thus, molecular analysis of blood or bone marrow might be useful to identify patients at increased risk of detrimental outcomes, who could receive more intensive therapy and/or vigilant monitoring. A similar approach could be adopted for other histiocytic neoplasms. Mutation detection in blood or bone marrow can also be used to monitor response to therapy, assessing the disappearance or persistence of mutated cells or cfDNA [125, 126, 128, 214, 222, 223, 224, 225, 226, 227, 228, 229, 230]. Similarly, minimal residual disease is assessed in many other haematologic neoplasms, where it has already led to personalised care and improved outcomes [231, 232].

### Targeted therapy with kinase inhibitors

Treatment of histiocytosis has long evolved empirically, rather than through mechanistic insight [233]. Vinblastine, for example, was extracted from a subtropical plant in the 1950s to test its antidiabetic properties [234]. Although it had little effect on blood glucose levels, it induced leukopenia in rats. This unexpected effect led investigators to assess its activity in haematologic neoplasms, where it rapidly became the backbone of first‐line systemic therapy for childhood LCH [22, 23, 235, 236, 237]. The discovery of somatic activating mutations enabled the first rational therapy for histiocytosis [6]. Robust responses to BRAF, MEK and other kinase inhibitors have now been demonstrated in patients with diverse histiocytoses [59, 226, 238, 239, 240, 241, 242, 243], regularly within days of initiation. MEK inhibition can even be effective in cases without detected driver alterations [174, 242], highlighting the general dependence on MAPK signalling. However, when mutations activate multiple signalling pathways, such as receptor tyrosine kinase alterations, MEK inhibition may be insufficient. Accordingly, a patient with *CSF1R*‐mutated ECD did not respond to the MEK inhibitor cobimetinib but had a complete response to the CSF‐1R inhibitor pexidartinib [85]. RAF‐independent *MAP2K1* variants are sometimes also associated with resistance to MEK inhibition [244, 245], but respond to downstream ERK inhibition [202]. An important limitation of many available inhibitors is their limited CNS penetration. Vemurafenib, in particular, seems unable to prevent the development of neurodegenerative disease [174, 246, 247]. Thus, other inhibitors are being tested that have superior CNS penetration [248, 249, 250]. Finally, targeted therapy is often not capable of eradicating the disease in patients with LCH or ECD but rather induces clinical remission by rendering mutant cells static [251]. Although this prevents these cells from causing systemic inflammation and associated tissue damage [251], cessation of targeted therapy often results in quick relapse of the disease [174, 226, 240, 252]. Interestingly, treatment‐free remission has been achieved in some cases without *BRAF* mutations [252] and is remarkably common in patients with ALK‐positive histiocytosis who discontinue targeted therapy [253, 254]. Understanding why remission is sustained in these patients will be crucial for advancing therapeutic strategies. Currently, several studies explore combination strategies in LCH/ECD to eradicate the neoplastic clone and achieve a cure for these patients [214, 229, 255].

## Conclusions

Genomic discoveries have redefined histiocytosis as a spectrum of diseases unified by MAPK pathway activation yet diversified by genetic variation and distinct cellular origins. These insights have fundamentally changed histiocytosis diagnosis, risk stratification, disease monitoring and treatment—most notably through the advent of kinase inhibitors that have dramatically improved patient outcomes. At the same time, major questions remain—for the timing of oncogenic events and their impact on different progenitors to the interplay between neoplastic cells and their environment and what governs the persistence of mutated clones. Interdisciplinary, collaborative efforts will be essential to answer these questions and continue the remarkable progress of recent decades.

## Author contribution statement

PGK drafted the manuscript and prepared the figures and tables. AGSvH, TvW and PCWH provided supervision and revised the manuscript.

## Permission to reproduce material from other sources

Parts of this work are derived from the introduction and discussion of the PhD thesis *Genetic and cellular origins of histiocytic neoplasms* by Paul G. Kemps (ISBN 978‐94‐6522‐001‐7). Figure 3A is based on an illustration by Laurenti, *et al* [124]. Figure 4 is adapted from Calvanese, *et al* [188].

## Data Availability

Data sharing not applicable to this article as no datasets were generated or analysed during the current study.
